# Association of socioeconomic inequality and risk of periprosthetic joint infection after total knee arthroplasty: a Danish cohort study of 75,141 cases

**DOI:** 10.2340/17453674.2025.43678

**Published:** 2025-05-19

**Authors:** Marie ANNEBERG, Anders TROELSEN, Per GUNDTOFT, Alma B PEDERSEN

**Affiliations:** 1 Department of Clinical Epidemiology, Aarhus University/Aarhus University Hospital; 2 Department of Orthopedic Surgery, Aalborg University Hospital; 3 Department of Orthopedic Surgery, Hvidovre Hospital; 4 Department of Orthopedic Surgery, Aarhus University Hospital; 5 Department of Clinical Medicine, Aarhus University. Denmark

## Abstract

**Background and purpose:**

Awareness of socioeconomic disparities in outcomes following surgical procedures is increasing. This highlights a critical challenge for clinical practice and public health. We examined whether low socioeconomic position (SEP) was associated with the incidence of revisions due to periprosthetic joint infection (PJI) following total knee arthroplasty (TKA).

**Methods:**

This cohort study included 75,141 patients undergoing TKA (2010–2021), identified from the Danish Knee Arthroplasty Registry (DKR). Individual-level SEP information, including wealth, living arrangements, and education, was obtained from Danish social and administrative registries. Revisions due to PJI were identified using a method combining the DKR and microbiology data. We calculated the cumulative incidence of revision due to PJI at 90 days and 2 years, and 2-year hazard ratios (aHRs) of revision due to PJI for lower vs. higher SEP groups, adjusted for age, sex, weight, and Charlson Comorbidity Index scores, with 95% confidence intervals (CI).

**Results:**

The incidence of revision due to PJI after 2 years of follow-up was 1.5% (CI 1.3–1.6) for low-wealth patients vs. 1.2% (CI 1.1–1.3) for high-wealth patients (aHR 1.3, CI 1.1–1.5); 1.5% (CI 1.3–1.7) for patients living alone vs. 1.2% (CI 1.1–1.3) for those cohabiting (aHR 1.4, CI 1.2–1.6); and 1.3% (CI 1.1–1.4) for patients with low education vs. 1.2% (CI 1.0–1.4) for those with high education (aHR 1.0, CI 0.8–1.2).

**Conclusion:**

Revision due to PJI among low-wealth patients and those living alone versus the corresponding high-SEP group were associated with increased risk of revision due to PJI.

Periprosthetic joint infection (PJI) following total knee arthroplasty (TKA) is a severe complication associated with increased morbidity and mortality [1-3]. Identifying modifiable risk factors is crucial for improving outcomes in TKA patients [4-6].

Lower socioeconomic position (SEP) is generally associated with increased vulnerability to adverse health events, in part due to limited healthcare access, lifestyle-related risk factors, and environmental stressors [7]. The Centers for Disease Control and Prevention defines SEP as “A composite measure that typically incorporates economic, social, and work status” [8].

SEP contributes to healthcare disparities across various medical specialties, including orthopedics [9]. In a recent study, Edwards et al. demonstrated that low SEP adversely influences the risk of infection, including pneumonia, urinary tract infection, and PJI after total hip replacement [10]. In the United States, joint centers found that low income was a stronger predictor of lower satisfaction and poorer functional outcomes than demographic characteristics or implant-related factors among younger patients [11]. In contrast, Dekeyser et al. (2020) showed no significant correlation between income or education and PJI risk after total joint arthroplasty (TJA) but showed that Medicaid payer status was associated with a higher risk of PJI [8].

Using high-quality population based Danish medical, social, and administrative registries [12], we aimed to investigate the association between SEP factors, including wealth, living arrangement, and education level, and the incidence of revision due to PJI after TKA.

## Methods

### Setting, study design, and data sources

Denmark (population 5.9 million in 2024) offers tax-funded healthcare, providing services to all citizens and residents without out-of-pocket expenses at the point of care, ensuring access based on need rather than ability to pay. Denmark has a long tradition of maintaining comprehensive medical, social, and administrative registries, inter-linkable via a unique civil registration number, ensuring near-complete follow-up of all residents.

We linked data from the following registries:

1. The Danish Civil Registration System, which contains data on vital status, migration, age, and sex [13].
2. Statistics Denmark, which maintains socioeconomic data on all Danish residents. We obtained family income, family liquid assets, living arrangement, and educational level [14].
3. The Danish Knee Arthroplasty Registry (DKR), which contains data on all primary and revision TKAs performed in Danish orthopedic departments. Validated annually against the Danish National Patient Registry (see below), the DKR achieved registration completeness of 92% for primary arthroplasties and 89% for revisions in 2010, improving to 99% and 98%, respectively, by 2021. We included details on surgery type, side, indication, dates, revision cause, patient weight, and height [15].
4. The Danish National Patient Registry (DNPR), which contains data on all hospital admissions and outpatient visits with diagnoses and procedures coded using the International Classification of Diseases (ICD). We linked information on diabetes mellitus (hereafter referred to as diabetes), psychiatric diseases, and comorbidities assessed via the Charlson Comorbidity Index (CCI), wherein a higher score indicates more comorbidity, using a 10-year look-back period [16].
5. The Danish Microbiology Database (MiBa), which holds electronic copies of all microbiological sample reports since 2010. A novel method integrating DKR and MiBa data has permitted more accurate PJI detection [17,18].

### Cohort

The study cohort included patients undergoing primary TKA from January 1, 2010, to November 21, 2021, identified in the DKR. In cases in which a patient had TKA on both knees, the 2 surgeries were treated as distinct cases. End of study date was November 21, 2023.

### Exposure

To examine SEP factors [8], we collected individual-level data on wealth (family income and family liquid assets), living arrangement, and education. Wealth was determined using either family income (total in a co-housing family before taxes) or family liquid assets (including cash property value, bank deposits, and securities such as stocks, bonds, and mortgage deeds), depending on the patient’s age. For patients aged < 65 years, family income was used. For patients aged ≥ 65 years, the standard retirement age in Denmark, family liquid assets were used, as income may no longer accurately reflect financial status. To account for annual variations, both measures were averaged over the 5 years preceding the primary TKA. Family income and liquid assets were each categorized into tertiles (low, medium, and high) for their age and combined into a single wealth variable [19,20]. Living arrangement was classified as living alone or cohabiting with an adult partner (married, unmarried, or living in multifamily housing). Educational levels were categorized based on highest completed education, as follows: low (none or elementary school), medium (more than elementary school, but less than university education), and high (university education) [8].

### Revision due to PJI

The study outcome was defined as revision due to PJI, identified either through registration in the DKR or via an algorithm combining microbiological data from MiBa and procedure codes from the DNPR [18]. This integrated approach enhanced detection of revision due to PJI, addressing the historical challenge of underreporting in arthroplasty registries when microbiology data is unavailable. This method identifies 42% more cases of revision due to PJI by capturing cases that may have been missed in the DKR, significantly improving PJI case identification compared with using the DKR alone.

Cases of revision due to PJI were identified in the DKR when a TKA was reported as undergoing revision due to deep infection, either “verified by microbiology” or “suspected.” If other of the possible causes of revision (aseptic loosening, pain without loosening, instability, secondary insertion of patellar component, wear of polyethylene, progression of osteoarthritis, other [seldom used]) were reported in the DKR alongside PJI, PJI was considered the main reason for revision.

A revision due to PJI was identified based on a combination of registration in the DKR and microbiology data when a TKA (linked between the DKR and DNPR via the personal identification number and primary surgery date ±14 days) was identified with a subsequent revision same-side procedure coinciding with a minimum of 2 positive microbiology tests yielding the same microorganism. We have described this method in detail in a previous publication [18]. These criteria align with the international definition of PJI [21].

### Statistics

We presented the patients’ characteristics as counts and percentages for categorical data and as medians with interquartile range (IQR) for continuous data. In addition, stacked bar plots were used to display patient characteristics. The density of exposure variables was analyzed using density graphs, in which all combinations of living arrangement and education levels were plotted against family income as a continuous variable. A density graph was used to visualize the distribution of data, showing where values were concentrated.

We followed the patients from the date of primary TKA until the date of the first revision due to PJI, other revisions, death, emigration, or the end of the study, whichever occurred first.

Cumulative incidences within 90 days and 2 years of the primary TKA date were calculated with 95% confidence intervals (CIs) using the Aalen–Johansen method [22], considering non-PJI revisions and death as competing risks. The results are presented graphically for each socioeconomic factor. We calculated the numbers needed to treat (NNT) as the reciprocal of the risk difference between groups with the lowest and highest SEPs.

We used a Cox regression model to calculate both crude and adjusted hazard ratios (HRs and aHRs) within 2 years of the primary TKA, with 95% CIs, comparing patients with low SEP (i.e., with low or medium wealth, living alone, only primary or secondary education) to patients with high SEP (i.e., high wealth, cohabitant, high education), while adjusting for age, sex, weight, and CCI score as potential confounders. The assumption of proportional hazards between groups was tested using log-minus-log plots and Schoenfeld residuals.

In sensitivity analyses, we stratified cumulative incidences by detailed living arrangement, including men and women living alone, married and unmarried cohabitants, and those in multi-family housing. We also assessed 2-year cumulative incidences and aHRs for men living alone vs. cohabiting men.

This study adheres to STROBE guidelines [23]. All analyses were conducted using R (R Foundation for Statistical Computing, Vienna, Austria).

### Ethics, data sharing plan, funding, use of AI, and disclosures

Ethical approval was not required for this non-interventional study, which was registered with the Danish Data Protection Agency (AU-2016-051-000001, Seq. No. 880). In preparing this manuscript, we used ChatGPT and Grammarly to assist in improving the grammar, language, and readability of the text. Complete disclosure of interest forms according to ICMJE are available on the article page, doi: 10.2340/17453674.2025.43678

## Results

### Patients’ characteristics

**Figure 1:**
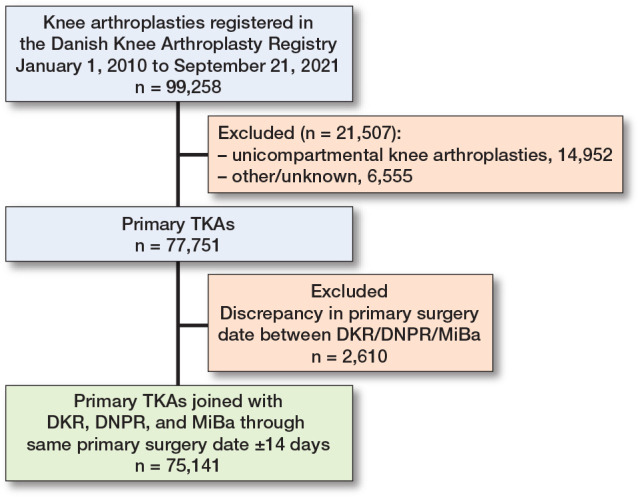
Study flowchart. DKR = Danish Knee Arthroplasty Registry; DNPR = Danish National Patient Registry; MiBa = Danish Microbiology Database; TKA = total knee arthroplasty.

**Figure 2:**
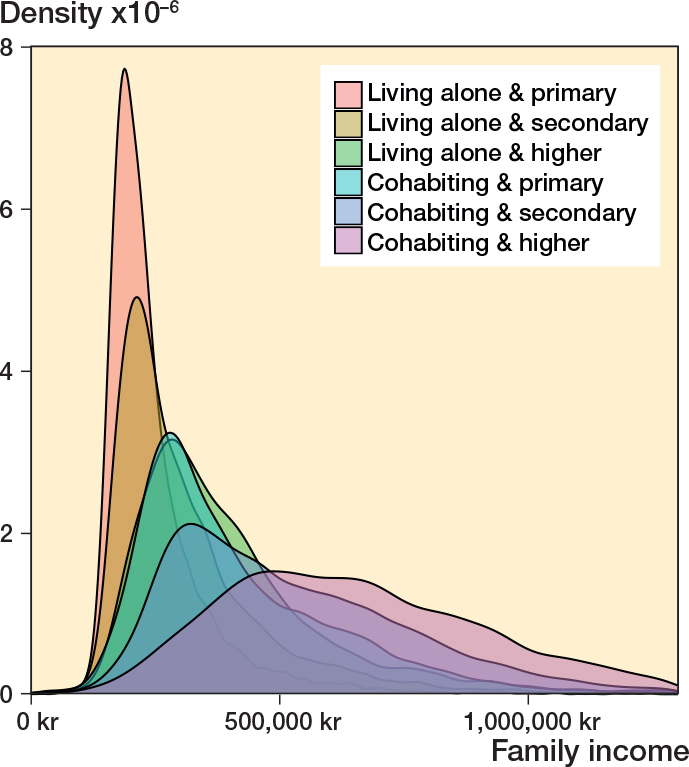
Density plot illustrating the distribution of income by combined education and cohabitation status. The density plot shows the distribution and concentration of data points. The area under each curve summariz-es to 1. TKA = total knee arthroplasty.

The observational cohort study encompassed 75,141 TKA surgeries (63,493 unique patients) (Figure 1). Information on SEP factors was available for nearly all patients (99% for wealth, 100% for living arrangement, and 97% for education). The density for living arrangement, education, and family income reflects the complex relationship between SEP factors. Cohabiting and better-educated patients generally have higher family incomes, while those living alone and with lower levels of education are more often concentrated at the lower end of the income scale (Figure 2).

**Figure 3:**
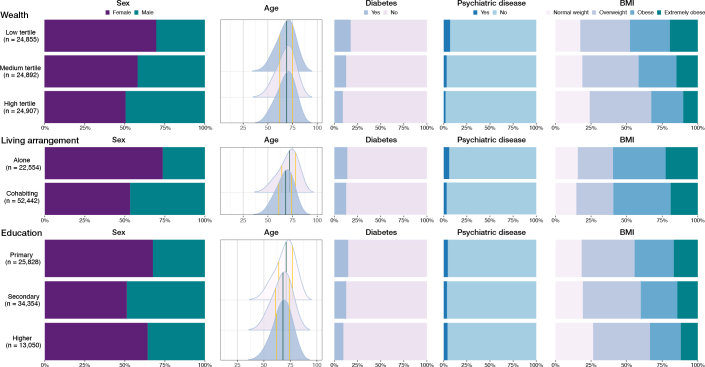
Demographic distributions of patient characteristics in the socioeconomic exposure groups. BMI = body mass index, n = number of cases.

Patients’ characteristics varied across categories of SEP factors. The indication for primary TKA was more likely to be rheumatoid arthritis in the lower-wealth group compared with the higher-wealth group (2.3% vs. 1.9%), but stable at 2% across living arrangements and education levels. The proportion of males ranged from 26% (living alone) to 49% (high wealth and secondary education). Diabetes prevalence was highest in the low-wealth group (18%) and lowest in the high-wealth (9%) and high-education groups (10%). Psychiatric disorders ranged from 2% (among high-wealth patients) to 7% (among patients who were both low-wealth) and 6% among patients living alone. Psychiatric disorders were at nearly the same level in the highest and lowest education groups, but the prevalence of affective disorders (mild to severe depression, bipolar disorders) was higher in the high-education group (not shown). Obesity or extreme obesity was most prevalent in the low-wealth and low-education groups (55% and 45%, respectively) and less common in the high-education group (34%) (Figure 3 and Table 1, see Appendix).

**Table 1:** Demographic distributions between socioeconomic groups. Values are count (%) unless otherwise specified

Factor Total (missing) Characteristic	Wealth tertile 74,654 (487)			Cohabitation 74,996 (145)		Education 73,232 (1,909)		
	Low	Medium	High	Living alone	Cohabiting	Primary	Secondary	Higher
Patients, n	24,855	24,892	24,907	22,554	52,442	25,828	34,354	13,050
Reason for primary TKA								
Primary osteoarthritis	21,246 (85)	21,039 (85)	20,624 (83)	19,456 (86)	43,731 (83)	22,296 (86)	28,584 (83)	10,777 (83)
Rheumatoid arthritis	579 (2.3)	568 (2.3)	465 (1.9)	526 (2.3)	1,090 (2.1)	568 (2.2)	721 (2.1)	291 (2.2)
Other	3,030 (12)	3,285 (13)	3,818 (15)	2,572 (12)	7,621 (15)	2,964 (12)	5,049 (15)	1,982 (15)
Age at primary TKA ^a^	69 (62–75)	69 (62–75)	69 (62–75)	72 (64–78)	68 (61–74)	71 (64–77)	68 (61–74)	68 (62–74)
0–59	5,306 (21)	5,309 (21)	5,314 (21)	3,809 (17)	12,244 (23)	4,602 (18)	8,221 (24)	2,837 (22)
60–69	8,588 (35)	8,595 (35)	8,599 (35)	6,114 (27)	19,790 (38)	7,638 (30)	12,663 (37)	5,082 (39)
70–79	8,722 (35)	8,741 (35)	8,746 (35)	8,918 (40)	17,369 (33)	10,266 (40)	11,162 (32)	4,239 (32)
≥ 80	2,239 (9.0)	2,247 (9.0)	2,248 (9.0)	3,713 (16)	3,039 (5.8)	3,322 (13)	2,308 (6.7)	892 (6.8)
Male sex	7,530 (30)	10,433 (42)	12,309 (49)	5,943 (26)	24,503 (47)	8,386 (32)	16,753 (49)	4,663 (36)
Weight kg ^a^	85 (74–97)	85 (74–97)	84 (74–95)	81 (70–94)	85 (75–97)	83 (73–95)	85 (75–98)	83 (72–95)
Body mass index category								
Under-/normal w. (< 25)	3,643 (17)	4,016 (19)	5,316 (24)	4,361 (23)	8,663 (19)	3,951 (18)	5,767 (19)	3,057 (26)
Overweight (25–30)	5,862 (28)	5,672 (27)	4,960 (22)	7,161 (37)	18,242 (40)	8,006 (37)	12,248 (41)	4,629 (40)
Obese (30–35)	7,308 (35)	8,428 (40)	9,561 (43)	4,788 (25)	11,791 (26)	5,921 (28)	7,730 (26)	2,503 (22)
Extremely obese (> 35)	4,116 (20)	3,200 (15)	2,261 (10)	3,022 (16)	6,607 (15)	3,630 (17)	4,315 (14)	1,383 (12)
Charlson Comorbidity Index score category								
Low (0)	2,373 (9.5)	1,834 (7.4)	1,509 (6.1)	2,010 (8.9)	3,711 (7.1)	2,230 (9)	2,501 (7.3)	855 (6.6)
Medium (1–2)	14,310 (58)	15,777 (63)	16,595 (67)	13,368 (59)	33,573 (64)	15,517 (60)	21,820 (64)	8,541 (65)
High (≥ 3)	8,172 (33)	7,281 (29)	6,803 (27)	7,176 (32)	15,158 (29)	8,081 (31)	10,033 (29)	3,654 (28)
Specific comorbidities								
Diabetes	4,391 (18)	3,164 (13)	2,280 (9.2)	3,162 (14)	6,716 (13)	3,853 (15)	4,403 (13)	1,273 (10)
Psychiatric disease	1,711 (6.9)	792 (3.2)	505 (2.0)	1,343 (6.0)	1,676 (3.2)	1,156 (4.5)	1,223 (3.6)	545 (4.2)
Wealth in tertiles for age ^b^								
Low	–	–	–	12,628 (56)	12,227 (23)	11,224 (44)	10,063 (29)	2,584 (20)
Medium	–	–	–	5,967 (27)	18,925 (36)	8,949 (35)	12,099 (35)	3,481 (27)
High	–	–	–	3,850 (17)	21,057 (40)	5,597 (22)	12,079 (35)	6,930 (53)
Living arrangements								
Living alone	12,628 (51)	5,967 (24)	3,850 (15)	–	–	9,320 (36)	8,922 (26)	3,732 (29)
Cohabiting	12,227 (49)	18,925 (76)	21,057 (85)	–	–	16,508 (64)	25,432 (74)	9,318 (71)
Education								
Primary	11,224 (47)	8,949 (36)	5,597 (23)	9,320 (42)	16,508 (32)	–	–	–
Secondary	10,063 (42)	12,099 (49)	12,079 (49)	8,922 (41)	25,432 (50)	–	–	–
Higher	2,584 (11)	3,481 (14)	6,930 (28)	3,732 (17)	9,318 (18)	–	–	–

a Values are mean (interquartile range)

b Family income for patients < 65 years, liquid assets for patients ≥ 65. Patients are divided into tertiles for their age.

PJI = periprosthetic joint infection, TKA = total knee arthroplasty.

### Socioeconomic factors and revision due to PJI

**Figure 4:**
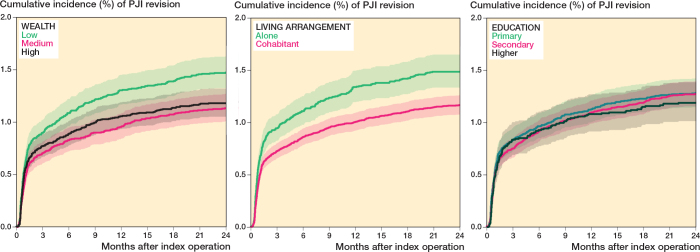
Cumulative incidences of revision due to periprosthetic joint infection (PJI) in the socioeconomic position groups.

The overall cumulative incidence of revision due to PJI was 0.8% (CI 0.7–0.9) after 90 days of follow-up and 1.3% (CI 1.2–1.4) 2 years after TKA. After 2 years, the cumulative incidences of the competing risks of aseptic revision, emigration, and death were 1.8% (CI 1.7–1.9), 0.1% (CI 0.1–0.1), and 1.9% (CI 1.8–2.0), respectively. The cumulative incidence of revision due to PJI in the wealth group was 0.9% (CI 0.8–1.0) after 90 days and 1.5% (CI 1.3–1.6) after 2 years for patients in the low tertile, compared with 0.8% (CI 0.7–0.9) after 90 days and 1.2% (CI 1.1–1.3) after 2 years for those in the high tertile. We also observed a higher incidence of revision due to PJI among persons living alone, with risks of 0.9% (CI 0.8–1.1) after 90 days and 1.5% (CI 1.3–1.7) after 2 years, compared with 0.7% (CI 0.7–0.8) after 90 days and 1.2% (CI 1.1–1.3) for cohabitants. Cumulative incidences were similar across education subgroups, aligning with the overall incidence (Table 2, Figure 4). The NNT was 333 (CI 200–1,000) for individuals in the low-wealth group vs. the high-wealth group, and 333 (CI 200–1,000) for patients living alone vs. cohabiting patients.

**Table 2:** Cumulative incidences and adjusted hazard ratios for revision due to PJI after total knee arthroplasty

Factor	Cumulative incidence (%) (CI)		aHR (CI) ^a^ 2 years
	90 days	2 years	
Overall			
Revision due to PJI	0.8 (0.7–0.9)	1.3 (1.2–1.4)	–
Aseptic revision	0.2 (0.2–0.3)	1.8 (1.7–1.9)	–
Emigration	0.0 (0.0–0.0)	0.1 (0.1–0.1)	–
Death	0.2 (0.2–0.3)	1.9 (1.8–2.0)	–
Wealth tertile			
Low	0.9 (0.8–1.0)	1.5 (1.3–1.6)	1.3 (1.1–1.5)
Medium	0.7 (0.6–0.8)	1.1 (1.0–1.3)	1.0 (0.8–1.1)
High	0.8 (0.7–0.9)	1.2 (1.1–1.3)	Reference
Cohabitation			
Living alone	0.9 (0.8–1.1)	1.5 (1.3–1.7)	1.4 (1.2–1.6)
Cohabitant	0.7 (0.7–0.8)	1.2 (1.1–1.3)	Reference
Education			
Primary	0.8 (0.7–0.9)	1.3 (1.2–1.4)	1.0 (0.8–1.2)
Secondary	0.8 (0.7–0.8)	1.3 (1.2–1.4)	1.0 (0.8–1.2)
Higher	0.8 (0.7–1.0)	1.2 (1.0–1.4)	Reference

a aHR = adjusted hazard ratio, adjusted for sex, age, bodyweight, and Charlson Comorbidity Index score.

PJI = periprosthetic joint infection; CI = 95% confidence interval.

The adjusted HRs for revision due to PJI within 2 years after TKA, accounting for sex, age, weight, and CCI score, were 1.3 (CI 1.1–1.5) for low-wealth vs. high-wealth patients, 1.4 (CI 1.2–1.6) for patients living alone vs. those cohabiting, and 1.0 (CI 0.8–1.2) for patients with low education vs. those with high education.

**Figure 5:**
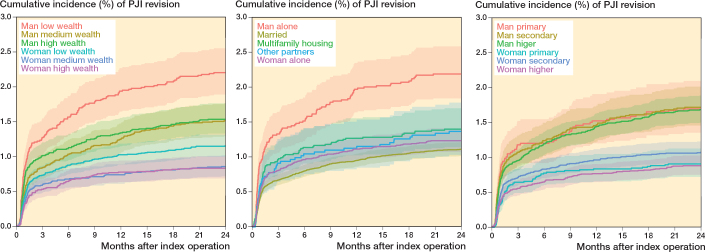
Cumulative incidences of revision due to periprosthetic joint infection (PJI).

Cumulative incidence graphs showed that men had a higher risk of revision due to PJI than women. Among men, those in the lowest wealth tertile had a significantly higher risk compared with those in the middle and highest tertiles. Regarding living arrangements, men living alone had twice the risk of revision due to PJI compared with women living alone, whereas married men and women had a lower risk than their unmarried counterparts. In terms of education, a marked sex difference in PJI risk was observed, but no significant variation was found within each sex across different education levels (Figure 5).

The cumulative incidence of PJI among men living alone was 2.2% (CI 1.8–2.6) after 2 years of follow-up with an aHR of 1.3 (CI 1.1–1.6) relative to cohabiting men, among whom the cumulative incidence was 1.6% (CI 1.4–1.7), resulting in an NNT of 125 (CI 100–500) (Table 3, see Appendix).

**Table 3:** Number-needed-to-treat at 2 years in socioeconomic groups: sensitivity analyses of cumulative incidence and adjusted HR for men living alone vs. men cohabiting

Factor	Cumulative incidence (CI)	Adj HR ^a^ (CI)	Risk difference, % (CI) (low vs. high in group)	NNT 100/RD (CI)
Overall	1.3 (1.2–1.4)			
Wealth tertile				
Low	1.5 (1.3–1.6)	1.3 (1.1–1.5)	0.3 (0.1 to 0.5)	333 (200–1,000)
Medium	1.1 (1.0–1.3)	1.0 (0.8–1.1)		
High	1.2 (1.1–1.3)	Reference		
Cohabitation				
Living alone	1.5 (1.3–1.7)	1.4 (1.2–1.6)	0.3 (0.1 to 0.5)	333 (200–1,000)
Cohabitant	1.2 (1.1–1.3)	Reference	–	
Men living alone	2.2 (1.8–2.6)	1.3 (1.1–1.6) ^b^	0.8 (0.2 to 1.0) ^c^	125 (100–500)
Men cohabiting	1.6 (1.4–1.7)	Reference	–	
Education				
Primary	1.3 (1.2–1.4)	1.0 (0.8–1.2)	0.1 (–0.1 to 0.3)	1,000 ^d^
Secondary	1.3 (1.2–1.4)	1.0 (0.8–1.2)		
Higher	1.2 (1.0–1.4)	Reference		

a Adjusted for sex, age, bodyweight, and Charlson Comorbidity Index score.

b Adjusted for age, bodyweight, and Charlson Comorbidity Index score.

c Vs. all men cohabiting.

d Undefined NNT confidence interval due to negative lower bound of RD confidence interval. When the RD is negative, the concept of NNT is not applicable as it implies harm rather than benefit.

CI = 95% confidence interval; NNTI = number-needed-to-treat; PJII = periprosthetic joint infection.

## Discussion

We aimed to examine SEP factors and the incidence of revision due to PJI after TKA in a large cohort study in a country providing tax-funded free access to healthcare. The risk of PJI within 2 years was higher among patients with low wealth (vs. high wealth), and among patients living alone (vs. cohabiting). The association between education and revision due to PJI was less clear.

Across all analyses, men had a higher risk of revision due to PJI than women, reinforcing well-established knowledge of sex differences in PJI risk [4,5]. However, the differences observed in the sex-stratified analyses within each SEP group, such as the elevated risk among men with low wealth and those living alone, suggest that SEP independently contributes to the observed differences, beyond the effect of sex alone. Our findings align with a Danish study on total hip arthroplasty patients, which identified an elevated 90-day infection risk (including pneumonia, urinary tract infection, and PJI) among individuals with low income (vs. high income) and those living alone (vs. those cohabiting) [10]. Additionally, they reported higher infection risks among individuals with low education (compared with high) and limited savings (vs. greater savings). In the same cohort, the study group also found that patients with low income (vs. high income) and those living alone (vs. cohabiting) were at elevated risk of any revision surgery and mortality after total hip arthroplasty [20].

Our current study identified an elevated PJI risk in the low-wealth group compared with the high-wealth group. This difference could be partly due to differences in patient characteristics (including indication for primary TKA rheumatoid arthritis, male sex, high BMI, and more comorbidities). This difference should be considered within the context of Danish society, which is relatively homogeneous in terms of income distribution, in contrast to the greater income inequality observed in countries like the United States and the United Kingdom [24]. Our classification of the wealth variable into tertiles may also have contributed to the small observed difference in revision due to PJI risk and rate. SEP is a complex multifaceted concept that cannot be fully captured by splitting patients into groups, particularly for continuous variables like income and liquid assets.

We found that patients living alone had a higher risk of PJI. Previous studies have shown that living without social support negatively impacts patient-reported outcomes after total joint replacement, and that social support provides psychological and material resources that protect overall health [25].

Although living arrangements are not easily modified, intermediate factors such as lack of self-efficacy, poor confidence in mobilization, and reluctance to seek medical care for early signs of infection may influence their effect on PJI risk.

Men living alone (vs. women living alone) or men with low wealth (vs. others), have a particularly higher risk of revision due to PJI. This elevated risk of revision due to PJI among men may be due to factors such as poorer health behaviors, a greater burden of comorbidities, poorer diet, and higher rates of smoking and alcohol consumption [4,5,24,26]. Unfortunately, we lacked data on these lifestyle factors. We adjusted our analysis using the CCI score as a proxy. However, the CCI only captures certain diagnoses from hospital contacts and excludes diagnoses managed in general practices and non-CCI comorbidities.

Our analysis revealed no significant differences in the association between education level and revision due to PJI. Education is an indicator of both the long-term effects of early life circumstances on adult health and the impact of adult resources on health status, and tends to remain stable lifelong. The education level of members of this study population, with an average age of approximately 70 years, reflects the educational context of approximately 50 years ago. At that time education may not have had the same impact on SEP as it does today. Moreover, the classification of education into low, medium, and high categories may not accurately reflect perceptions of these levels at that time. For example, gymnasium (equivalent to high school in the USA), which is now considered a secondary level of education, may have been regarded as a higher level of education in the past. The minimal effect of education on the risk of PJI might also be partially explained by the generally lower PJI risk observed in women [5]. Over the past several decades, educational opportunities have expanded significantly across all societal groups [3]. As a result, women may be disproportionately represented among those with lower education levels due to historical sex disparities in education, potentially leading to an underestimation of the observed association.

The observed socioeconomic inequality in PJI risk in this Danish study is unrelated to healthcare affordability. Instead, it may instead reflect differences in health behaviors—such as smoking, alcohol use, poor diet, and physical inactivity. These behaviors tend to have strong social patterns, with unfavorable behaviors more common in lower SEP groups, potentially due to financial constraints and limited resources [27].

In the wealth and cohabitation groups, the NNT was 333 (CI 200–1,000), meaning that if a perfect intervention could eliminate the risk difference between living alone vs. cohabiting or high vs. low wealth, 333 TKA patients would prevent 1 revision due to PJI. However, among men living alone, the NNT was 125 (CI 100–500), indicating that in this subgroup, 1 case of revision due to PJI could be prevented for every 125 patients through the implementation of an effective intervention. This highlights the potential value of targeted interventions tailored to this at-risk group. Such intervention could include focus on reducing smoking and alcohol, and improving hygiene habits, and nutrition—both pre- and post-surgery. Additionally structured follow-up care with proactively scheduled clinic visits in the months following surgery to evaluate mobilization and wound healing, rather than relying solely on patient-initiated contacts, could further mitigate PJI risk. Such tailored strategies may enhance early detection and intervention, potentially improving postoperative outcomes among men who live alone.

### Strengths

The strengths of this study include its use of prospective data collection, whereby individual-level information on SEP factors was collected from Danish population-based health, social, and administrative registries. This minimized risk of selection bias, as all TKA patients treated in public and private hospitals were included. We included wealth, living arrangements, and education as SEP factors, with nearly complete information on all patients.

Another strength is the definition of revision due to PJI. In a previous study, we developed and validated a method for identifying PJI by combining DKR and microbiological data. This method enhances the capture of revision due to PJI, limiting previous problems of underestimating PJI when using national arthroplasty registries alone [18]. Although this method is not perfect and may still carry a minor risk of underestimating PJI, we believe it has not affected the observed associations in our study.

### Limitations

We lack data on lifestyle factors (smoking, alcohol use, diet, and physical activity), which would have helped assess their distribution across SEP groups. Although we adjusted for age, sex, weight, and CCI score, as proxies, the absence of lifestyle data limits our ability to evaluate the role of these lifestyle factors as explanatory variables. This limitation may have led to unmeasured confounding by lifestyle factors and could have partly explained the observed association between lower SEP and increased PJI risk, potentially overstating the strength of SEP as an independent predictor. However, it is also possible that some lifestyle factors function as intermediate variables in the causal pathway between SEP and PJI, rather than as confounders, and that adjusting for them could attenuate the effect of SEP that we aim to evaluate.

The external validity of this study may be limited to similar healthcare settings, as SEP varies across countries due to differences in cultural norms, economic structures, and healthcare systems.

### Conclusion

Low wealth and living alone, particularly among men, were the SEP factors most strongly associated with revision due to PJI after TKA. Lifestyle factors may contribute to these associations.

*In perspective,* our findings highlight the importance of attention to patients’ SEP, to help identify modifiable risk factors both pre- and postoperatively, to potentially reduce the risk of revision due to PJI.

### Supplementary data

Codes used in this study are available as supplementary data on the article page, doi: 10.2340/17453674.2025.43678

## Supplementary Material



## Notes

MA managed, linked, analyzed the data, and drafted the manuscript. All authors participated in the design of the study, interpretation of the results, and approved the final manuscript.

Henrik Toft Sørensen helped in reviewing and editing of the manuscript

Handling co-editors: Marianne Westberg and Philippe Wagner

*Acta* thanks Håvard Dale and Anna Stefánsdóttir for help with peer review of this manuscript.
