# Supplementary material for: Association of socioeconomic inequality and risk of periprosthetic joint infection after total knee arthroplasty: a Danish cohort study of 75,141 cases
Source: Acta Orthop. 2025 May 19;96:371–9. doi: 10.2340/17453674.2025.43678 (PMC12087143; doi:10.2340/17453674.2025.43678)
Supplement: Supplementary file 1 [file ActaO-96-43678-s1.pdf]

# Supplementary data

## Codes used in the study

| Description | Coding system | Data source |
|---|---|---|
| **Study Population** | | |
| Primary TKA surgery in a public or private hospital in Denmark during the study period from 1 January 2010 to 21 September 2021, as registered in the DKR. | | DKR |
| **Exposure** | | |
| Family income | | Statistics Denmark |
| Cohabiting status | | Statistics Denmark |
| Education level | | Statistics Denmark |
| **Covariates** | | |
| **Diabetes mellitus** | **ICD-10[a]** | |
| Diabetes mellitus | E10-E14 | DNPR |
| **Glucose-lowering medication** | **ATC[b]** | |
| Biguanides | A10BA | Prescription Database |
| Sulphonylureas | A10BB | Prescription Database |
| Thiazolidinediones | A10BG | Prescription Database |
| Meglitides | A10B | Prescription Database |
| Dipeptidyl peptidase-4 inhibitors | A10BH | Prescription Database |
| Insulin | A10A | Prescription Database |
| Glucagon-Like Peptide-1 Receptor Agonist | A10BJ | Prescription Database |
| Sodium-Glucose cotransporter-2 Inhibitors | A10BK | Prescription Database |
| **Psychiatric disease** | **ICD-10[a]** | |
| Organic, including symptomatic, mental disorders | F00-F03, G30 | DNPR |
| Mental and behavioral disorders due to psychoactive substance use | F10-F13, F19 | DNPR |
| Schizophrenia, schizotypal, and delusional disorders | F20-F29 | DNPR |
| Mood [affective] disorders | F30, F31, F322, F323, F332, F333, F320, F321, F328, F329, F330, F331, F338, F339, F34, F38, F39 | DNPR |
| Neurotic, stress-related, and somatoform disorders | F40 - F45, F48 | DNPR |
| Behavioral syndromes associated with physiological disturbances and physical factors | F500, F502, F505, F509 | DNPR |

| | | |
|---|---|---|
| Disorders of adult personality and behavior | F60-F61 | DNPR |
| Intellectual disability | F70-F79 | DNPR |
| Disorders of psychological development | F82, F84 | DNPR |
| Behavioral and emotional disorders with onset usually occurring in childhood and adolescence | F90, F98-8, F98-9 | DNPR |
| Any mental disorder | F00-F98 | DNPR |
| **Outcome: PJI (and any revision)** | | |
| **In the DKR a revision is defined as any later procedure that involves a supplement to, exchange, removal, or modification of an already existing arthroplasty.** | | |
| **Revision due to PJI in the DKR:** | | |
| Deep infection (verified by microbiology) | | DKR |
| Deep infection (suspected) | | DKR |
| **Any revision as recorded in the DKR** | | |
| Aseptic loosening | | DKR |
| Pain without loosening | | DKR |
| Instability | | DKR |
| Deep infection (verified by microbiology) | | DKR |
| Deep infection (suspected) | | DKR |
| Secondary insertion of patella component | | DKR |
| Wear of polyethylene (patella) | | DKR |
| Wear of polyethylene (tibia) | | DKR |
| Progression of osteoarthritis | | DKR |
| Other (seldom used) | | DKR |
| **From: the DNPR/MiBa in combination** | | |
| **PJI: A combination of “any revision” from the DNPR AND 2 positive samples from MiBa** | | |
| **Any revision from the DNPR** | **NOMESCO** | |
| Open exploration of soft tissues in the knee | KNGA02A | DNPR |
| Open exploration of the knee joint | KNGA12 | DNPR |
| Open joint biopsy in the knee | KNGA22A | DNPR |
| Open soft tissue biopsy in the knee | KNGA22C | DNPR |
| Secondary insertion of all components of uncemented total knee prosthesis | KNGC20 | DNPR |
| Secondary insertion of proximal components of uncemented total knee prosthesis | KNGC21 | DNPR |

| | | |
|---|---|---|
| Secondary insertion of distal components of uncemented total knee prosthesis | KNGC22 | DNPR |
| Secondary insertion, patellofemoral component, uncemented total knee prosthesis | KNGC23 | DNPR |
| Secondary insertion of uncemented total knee prosthesis, unspecified | KNGC29 | DNPR |
| Secondary insertion of all components of hybrid total knee prosthesis | KNGC30 | DNPR |
| Secondary insertion of proximal components of hybrid total knee prosthesis | KNGC31 | DNPR |
| Secondary insertion of distal components of hybrid total knee prosthesis | KNGC32 | DNPR |
| Secondary insertion, patellofemoral component, hybrid total knee prosthesis | KNGC33 | DNPR |
| Secondary insertion of hybrid total knee prosthesis, unspecified | KNGC39 | DNPR |
| Secondary insertion of all components of cemented total knee prosthesis | KNGC40 | DNPR |
| Secondary insertion of proximal components of cemented total knee prosthesis | KNGC41 | DNPR |
| Secondary insertion of distal components of cemented total knee prosthesis | KNGC42 | DNPR |
| Secondary insertion, patellofemoral component, cemented total knee prosthesis | KNGC43 | DNPR |
| Secondary insertion of cemented total knee prosthesis, unspecified | KNGC49 | DNPR |
| Secondary insertion of interpositional prosthesis in the knee joint | KNGC59 | DNPR |
| Other secondary insertion of knee joint prosthesis | KNGC99 | DNPR |
| Open total synovectomy in the knee joint | KNGF02 | DNPR |
| Open partial synovectomy in the knee joint | KNGF12 | DNPR |
| Resection arthroplasty in the knee | KNGG09 | DNPR |
| Interpositional arthroplasty in the knee | KNGG19 | DNPR |
| Other arthroplasty without prosthesis in the knee | KNGG29 | DNPR |

| | | |
|---|---|---|
| Arthrodesis with external fixation in the knee | KNGG59 | DNPR |
| Open release of adhesions in the knee joint | KNGH32 | DNPR |
| Other open knee joint surgery | KNGH92 | DNPR |
| Fenestration or perforation of bone in the knee/leg | KNGK29 | DNPR |
| Excision of a bursa in the knee/leg | KNGM79 | DNPR |
| Disarticulation of the knee joint | KNGQ09 | DNPR |
| Other amputation surgery on the knee/leg | KNGQ99 | DNPR |
| Incision and revision of infection in the knee joint | KNGS19 | DNPR |
| Incision/revision of bone infection in the knee/leg | KNGS29 | DNPR |
| Incision/revision with the installation of drugs for infection in the knee joint | KNGS49 | DNPR |
| Incision/revision/installation of drugs for bone infection in the knee/leg | KNGS59 | DNPR |
| Other operations for infection in the tendon, joint, or bone in the knee/leg | KNGS99 | DNPR |
| Removal of all components of total knee prosthesis | KNGU10 | DNPR |
| Removal of the medial part of total knee prosthesis | KNGU11 | DNPR |
| Removal of the lateral part of total knee prosthesis | KNGU12 | DNPR |
| Removal of total knee prosthesis, unspecified | KNGU19 | DNPR |
| Removal of another implant in the knee/leg | KNGU99 | DNPR |
| Reoperation for superficial infection after surgery on the knee/leg | KNGW59 | DNPR |
| Reoperation for deep infection after surgery on the knee/leg | KNGW69 | DNPR |
| Reoperation for deep hemorrhage after surgery on the knee/leg | KNGW89 | DNPR |
| Other reoperation after surgery on the knee/leg | KNGW99 | DNPR |
| **From: MiBa** | | |
| **Cultures were taken between 24 hours before revision surgery and 48 hours after revision surgery if a minimum of 2 samples out of a minimum of 3 samples were positive for the same microorganism. If only 1/5 of biopsies were positive or 2/5 were positive for different pathogens, contamination was considered to be present.** | | |
| **Samples considered from MiBa** | | |
| Tissue | | MiBa |
| Biopsy | | MiBa |
| Bone | | MiBa |
| Kamme Lindberg biopsies | | MiBa |
| Tissue from the joint capsule | | MiBa |

Abbreviations: ATC - Anatomical Therapeutic Chemical Classification System; DNPR - Danish National Patient Registry; DKR - Danish Knee Arthroplasty Registry; ICD - *International Classification of Diseases*; MiBa - Danish Microbiology Database; NOMESCO - The Nordic Medico-Statistical Committee procedure codes; PJI - Periprosthetic Joint Infection; TKA - Total Knee Arthroplasty.
